# The pattern of 1‐aminocyclopropane‐1‐carboxylate oxidase induction in the tomato leaf petiole abscission zone is independent of expression of the ribonuclease‐LX‐encoding *LeLX* gene

**DOI:** 10.1111/plb.12730

**Published:** 2018-04-26

**Authors:** M. Chersicola, A. Kladnik, M. Tušek Žnidarič, A. Lers, M. Dermastia

**Affiliations:** ^1^ Department of Biotechnology and Systems Biology National Institute of Biology Ljubljana Slovenia; ^2^ Jožef Stefan International Postgraduate School Ljubljana Slovenia; ^3^ Department of Biology Biotechnical Faculty University of Ljubljana Ljubljana Slovenia; ^4^ Department of Postharvest Science of Fresh Produce Agricultural Research Organization The Volcani Center Rishon LeZion Israel

**Keywords:** 1‐aminocyclopropane‐1‐carboxylate oxidase, abscission, ethylene, gene expression, localisation, RNase LX

## Abstract

- The abscission of tomato leaves occurs in the petiole abscission zone, and its late stage includes two spatially divided processes: cell separation and programmed cell death (PCD). Both of these processes are regulated by ethylene. The last step in ethylene biosynthesis is conversion of 1‐aminocyclopropane‐1‐carboxylic acid to ethylene, which is catalysed by the enzyme 1‐aminocyclopropane‐1‐carboxylate oxidase (ACO); however, the location of ACO in the leaf petiole abscission zone is not known. The tomato gene *LeLX* encodes ribonuclease LX, which is a marker for PCD and is induced by ethylene during abscission, but its association with ACO has not been explored.
- In a tomato transgenic line 1‐7 with inhibited expression of *LeLX* showing delayed leaf abscission, the morphology and ultrastructure of the leaf petiole abscission zone was examined. In this zone of the cv.’VF36’ and of a transgenic line 1‐7, spatiotemporal differences in expression of *LeACO1* and *LeACO4* were analysed and ACO protein was detected immunohistochemically.
- In comparison to wild‐type plants, there were no obvious morphological and ultrastructural features in the abscission zone of plants of a transgenic line 1‐7 before and after abscission induction. *LeACO1* expression was low before abscission induction, and increased 24 h after induction, although with no apparent spatial pattern. In contrast, *LeACO4* was expressed before abscission induction, and its transcript level declined 24 h after induction on the distal side of the abscission zone fracture. In the *LeLX*‐inhibited transgenic line, there were no significant differences in *LeACO1* and *LeACO4* expression in the petiole abscission zone, in comparison to wild‐type plants. In addition, the ACO protein was immunolocalised to the vascular tissues that traverse the petiole abscission zone in plants of wild type and of a transgenic line 1‐7; and additionally in the plane of future abscission zone fracture of transgenic‐line plants.
- The results suggest temporal differential expression of the *LeACO* genes in tomato leaf petioles and vascular localisation of ACO1 protein. Additionally, the results indicate that expression of *LeACO* genes is not affected by suppression of the *LeLX* expression.

## Introduction

Abscission is a process in plant development where leaves, flowers and fruits separate from the plant in a temporally and spatially regulated manner (Roberts *et al*. 2002; Leslie *et al*. 2007). It occurs specifically in the tissue of the preformed abscission zone (AZ), which is usually located at the base of the organ to be shed. It has been shown that abscission of the tomato leaf petiole and flower pedicel includes two late spatially divided processes: cell separation and programmed cell death (PCD; Bar‐Dror *et al*. 2011; Dermastia *et al*. 2012; Chersicola *et al*. 2017). Both of these processes are induced by ethylene (Trobacher 2009; Meir *et al*. 2010; Chersicola *et al*. 2017).

The last step in ethylene biosynthesis is the conversion of 1‐aminocyclopropane‐1‐carboxylic acid to ethylene, which is catalysed by the enzyme 1‐aminocyclopropane‐1‐carboxylate oxidase (ACO; Ruduś *et al*. 2013). ACO is encoded by a small multigene family (Ruduś *et al*. 2013; Seymour *et al*. 2013), and its expression is tightly linked to the amount of ethylene produced by the plant (Van de Poel *et al*. 2012; Ruduś *et al*. 2013). It has been shown that three *Arabidopsis* ACO genes are auto‐regulated by ethylene (De Paepe *et al*. 2004). Although ACO is a well‐characterised protein, its post‐translational regulation and combinatorial interplay are not well understood (Van de Poel & Van Der Straeten 2014).

Among seven *LeACO* genes (Seymour *et al*. 2013), *LeACO1* and *LeACO4* have their highest expression in tomato leaf and flower tissues (http://gbf.toulouse.inra.fr/tomexpress/www/welcomeTomExpress.php). We have recently demonstrated (Chersicola *et al*. 2017) that in the tomato flower pedicel, the expression of *LeACO1* and *LeACO4* is localised to the vascular tissues. Moreover, the ACO1 protein is mainly located in the cytoplasm of the phloem companion cells (Chersicola *et al*. 2017). However, it is not known where *LeACO* genes are expressed, and where the ACO protein is localised during abscission of the tomato leaf petiole.

It has been demonstrated that delay in tomato leaf abscission can occur if the gene encoding ribonuclease LX (RNase LX) is inhibited (Lers *et al*. 2006). Tomato RNase LX is a member of the T2/S‐like RNases (Lehmann *et al*. 2001). The *LeLX* gene that encodes RNase LX is an orthologue of *Arabidopsis RNS3*, which was recently linked to developmental PCD (Olvera‐Carrillo *et al*. 2015). *LeLX* expression has been detected at the distal side of the mature tomato leaf petiole abscission zone, where the RNase LX protein is localised and where prominent ultrastructural hallmarks of PCD have been observed (Bar‐Dror *et al*. 2011). However, it has not been demonstrated whether inhibition of LX is associated with PCD features in the tomato petioles.

In the present study, we re‐examined the abscission zone of a transgenic plant with inhibited LX, analysed the specific localisation of the expression of two tomato *LeACO* genes in the tomato leaf petiole AZ tissue, *LeACO1* and *LeACO4*, and also localisation of the ACO protein. In addition, we tested the hypothesis that inhibition of *LeLX* expression affects the expression of these ethylene biosynthetic genes.

## Material and methods

### Plant growth conditions and treatments

Wild‐type (WT) tomato plants (*Solanum lycopersicum* cv. ‘VF36’) and ‘VF36’ transgenic lines with modified *LeLX* expression, including the transgenic line 1‐7 that shows the highest inhibition of *LeLX* expression (Lers *et al*. 2006; Figure S1), were grown in growth chambers at 25 °C and 75% relative humidity, under a 16/‐h 8‐h day/night cycle. For induction of abscission, the leaf blades of up to four lower leaves were removed (*i.e*. debladed plants) using a sharp razor, while leaving most of the petiole intact. For acceleration of abscission, ethylene was applied to the debladed tomato plants 36 h later, by placing them in sealed containers with a 50 ppm ethylene atmosphere for 24 h. The control plants were not debladed, and were placed in sealed containers with a normal atmosphere for 24 h before sampling.

### Transmission electron microscopy

Samples were prepared exactly as in Bar‐Dror *et al*. 2011. In brief, at 24 h after the 24‐h ethylene treatment, the treated and untreated control plants were sampled. Blocks of tissue with the longest side measuring approximately 2 mm, containing the AZ were fixed in 3% (w/v) glutaraldehyde (in 0.08 M phosphate buffer, pH 7.2) and post‐fixed for 12 h in 1% (w/v) osmium tetroxide in the same buffer. Embedded (Agar 100 resin) samples were cut into ultrathin sections (70–90 nm) and stained in a 1% (w/v) water solution of uranyl acetate and water solution of lead citrate. Samples were observed with a CM 100 transmission electron microscope (Philips, Amsterdam, The Netherlands) operating at 80 kV and images were recorded with an ORIUS SC 200 camera using Digital Micrograph software (Gatan, Washington, DC, USA).

### Sampling and total RNA extraction

Thin layers of tissue were cut from each side of the leaf petiole AZ of the first three fully developed lower leaves of each tomato plant using a scalpel (*i.e*. leaves L1, L2, L3, with L1 as the lowest and oldest leaf on the stem). The samples were stored in plastic tubes and frozen in liquid nitrogen. The samples were homogenised in Tissuelyser (Qiagen, Hilden, Germany) and RNA was extracted using RNeasy Micro kits (Qiagen), according to manufacturer's protocol.

### Gene expression analysis

Gene expression assays for *LeACO1* and *LeACO4* (see Table 1) were designed using the Custom TaqMan Gene Expression Assays service (Applied Biosystems, Foster City, CA, USA). Expression assays and reagents (One‐Step RT‐PCR AgPath ID mastermix; Life Technologies, Carlsbad, CA, USA) were used according to the manufacturer's instructions. Quantitative reverse transcription real‐time PCR (qPCR) was performed in a final reaction volume of 5 μl, which contained 2 μl RNA and 3 μl reaction mix. The qPCR was performed using a real‐time PCR system and the default cycling conditions (ABI ViiA 7TM; Applied Biosystems). The relative expression of the target and reference genes was determined using the standard curve quantification method (Pfaffl 2006; Žel *et al*. 2012). An RNA pool of leaf petiole AZ samples was used to prepare a standard sample from which the standard calibration curve was constructed. Every sample was tested for each gene at two dilutions, and the relative copy numbers were calculated from the calibration curves. The expression of all of the genes was normalised relative to the expression of the reference gene that encodes cytochrome oxidase (*COX*; Bar‐Dror *et al*. 2011; Müller *et al*. 2015), and the gene expression levels are given as the relative numbers of copies.

**Table 1:** Primer and probe sequences for the *LeACO1* and *LeACO4* real‐time PCR assays

Gene name	Forward primer	Reverse primer	Probe	ITAG identifier
*LeACO1*	AAGGGACTCCGCGCTCATA	CAAGTTGGTCACCAAGGTTAACC	TCGATGTTCCTCCCATGCGCC	Solyc07 g049530
*LeACO4*	GGGAGAGCACTTTCTTCTTGAAACA	CTTCAGTGCAAAATCTTTCATAACTTTCCT	CAGGTCAGGAACTTCA	Solyc02 g081190

Two‐way anova was used to evaluate the differences in expression of the selected genes between the proximal and distal sides of the leaf petiole AZ, as sampled from the different leaves. For pair‐wise comparisons, Tukey *post‐hoc* tests were applied (*P *<* *0.05). All statistical analyses were performed using the custom scripts in the R software environment, version 3.1.2 (R Foundation for Statistical Computing, Vienna, Austria).

### Immunolocalisation

Immunolocalisation was performed as described in Chersicola *et al*. (2017). Parapalast Plus‐embedded tissue sections were placed on Superfrost Ultra Plus slides (Menzel‐Gläser). The sections were de‐waxed in xylene and rehydrated in a decreasing ethanol series, and finally in TBST (1 ×  TBS, pH 7.6, 0.2% Tween‐20). Antigen retrieval was performed by incubating the slides in sodium citrate buffer (10 mm sodium citrate, pH 6.0) for 15 min in a boiling water bath. The sections were incubated in blocking solution (TBST plus 5% normal donkey serum) for 30 min at room temperature, followed by incubation with the anti‐ACO1 primary antibody (aN‐19; 1:100 dilution; Santa Cruz Biotechnology, CA, USA) in blocking solution for 1 h at room temperature. The anti‐ACO1 polyclonal antibody used was raised in goat against a sequence between amino acids 1 to 50 of ACO of *A. thaliana* (accession# Q06588), which shares 82% identity with ACO1 of *S. lycopersicum*. The anti‐ACO1 specificity is shown in Figure S2. Non‐immune goat serum was used for the negative controls. After washing for 3 × 10 min in TBST, the sections were incubated with alkaline‐phosphatase‐conjugated anti‐goat antibodies (1:1000; Jackson ImmunoResearch, Carlsbad, CA, USA) in blocking solution for 1 h at room temperature, followed by washing as above. The staining was developed in nitro‐blue tetrazolium chloride/5‐bromo‐4‐chloro‐3‐indolyl‐phosphate substrate solution (Roche Diagnostics, Rische‐Rotkeuz, Switzerland), and the sections were washed with water, dehydrated rapidly through an increasing ethanol series, followed by xylene, and mounted in Permount (Electron Microscopy Sciences, Washington, PA, USA). The stained sections were observed under a microscope (Zeiss AxioImager Z1) and photographed in colour using a digital camera (AxioCam HRc; Zeiss, Oberkochen, Germany).

Immunolocalisation of TAPG4 was performed essentially as described above, with affinity purified anti‐TAPG4 polyclonal antibodies (BioGenes, Berlin, Germany) raised in rabbit against a peptide sequence 306‐320 (CPNHESCPNQGSGVK) of TAPG4 (accession# Q96488). The blocking solution was TBST plus 5% normal goat serum; the primary antibody was diluted 1:100 in blocking solution and the secondary antibody was alkaline‐phosphatase‐conjugated anti‐rabbit (1:1000, in blocking solution; Jackson ImmunoResearch). Washing and detection steps were performed as above.

## Results

### Differences in morphology and immunolocalisation of tomato abscission‐related polygalacturonase TAPG4 in the petiole abscission zone of WT and transgenic plants

**Figure 1:**
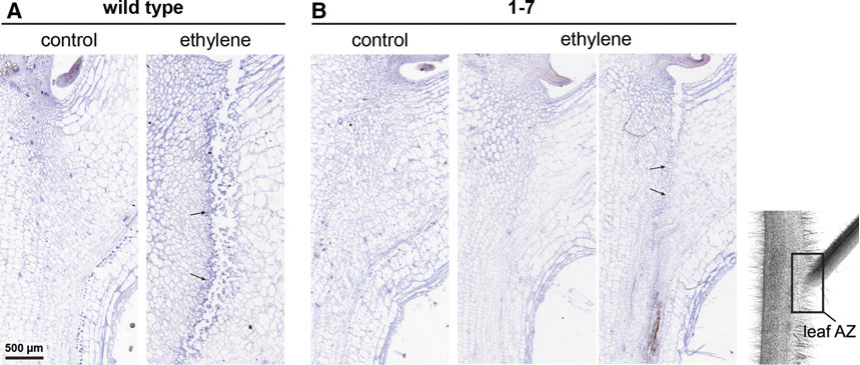
Immunolocalisation of the TAPG4 protein in the leaf petiole abscission zone (AZ) of ‘VF36’ tomato WT line (A) and transgenic line 1‐7 (B). Control, AZ before abscission induction; ethylene, AZ 24 h after abscission induction by leaf deblading and ethylene treatment. After 24 h of ethylene treatment the fracture in the WT line is complete, while in the transgenic line it is not formed or has just started. Bottom, right: Illustration of the leaf petiole AZ and location of the vascular tissue on the plant. Arrows indicate the presence of the immunolocalisation signal.

**Figure 2:**
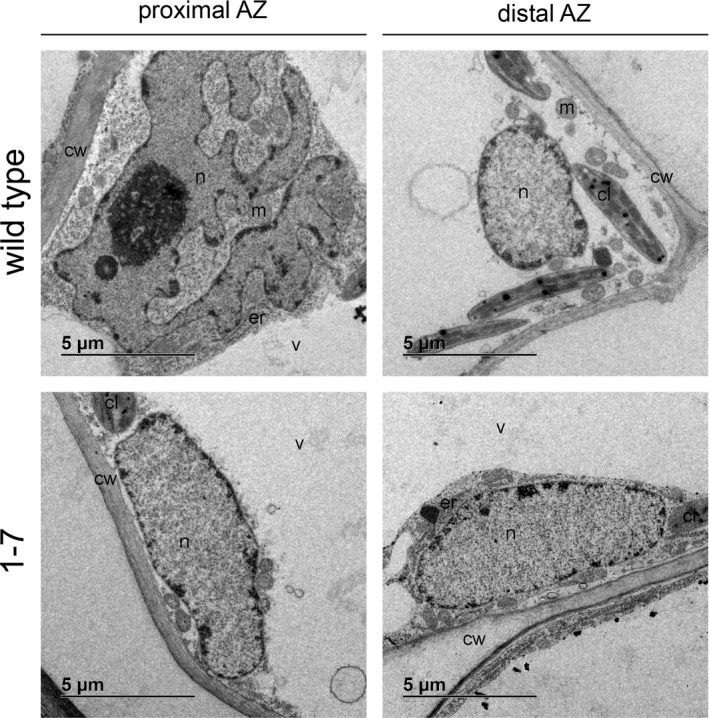
Transmission electron micrographs of cells in the tomato leaf petiole AZ of the ‘VF36’ tomato WT line and transgenic line 1‐7 showing ultrastructural changes 24 h after induction of abscission by leaf deblading and ethylene treatment. The proximal side of the AZ in the WT plants shows ameboidal nucleus as a hallmark of membrane trafficking (Bar‐Dror *et al*. 2011) and its distal side shows the features of PCD with ruptured tonoplast of the vacuole and other membrane organelles undergoing a stepwise degradation, but largely unprocessed dead cells (Bar‐Dror *et al*. 2011). The cells of the transgenic line cannot be differentiated from the cells of the AZ. Cell organelle labelling: cl, chloroplast; cw, cell wall; er, endoplasmic reticulum; m, mitochondria; n, nucleus; v, vacuole.

The petiole abscission zone of the WT plants before the abscission induction is morphologically distinct from neighbouring cells and includes smaller‐sized cells with dense cytoplasm (Fig. 1A). At 24 h after induction, in these plants the abscission was highly progressed with a complete fracture (Fig. 1A). On the other hand, the AZ was not pre‐formed before induction of abscission in plants of the transgenic line in which the abscission was delayed and the fracture started to form in only a few transgenic plants 24 h after abscission induction (Fig. 1B). While at that time point cells on the proximal side of the AZ of WT plants show hallmarks of high metabolic activity, there were no morphological changes observed in cells of the transgenic line (Fig. 2). However, similarly to WT plants, in plants of the transgenic line, a TAPG4 protein was localised to the cell row in which the fracture finally formed (Fig. 2). The prominent feature of the distal side of the WT petiole AZ after induction of abscission is PCD, associated with several ultrastructural hallmarks of the process (Fig. 2), (Bar‐Dror *et al*. 2011) and significant induction of *LeLX* on the distal side of the AZ (Figure S1). In contrast, no characteristic signs of PCD were revealed 24 h after abscission induction in the petiole abscission zone of the transgenic line (Fig. 2).

### Expression of *LeACO1* is increased after abscission induction with ethylene treatment

**Figure 3:**
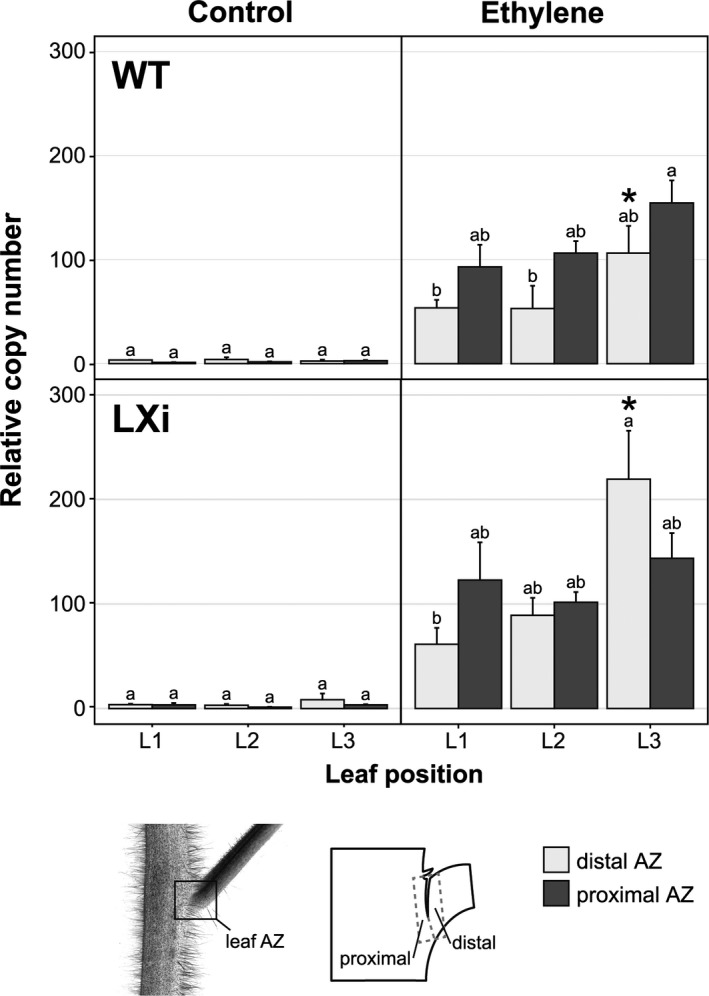
Gene expression profiles of *LeACO1* in the leaf petiole AZ of the ‘VF36’ tomato WT line and transgenic line 1‐7 (LXi) with suppressed *LeLX* expression. Control and ethylene‐treated samples of the distal (white) and proximal (grey) AZ were analysed, with the data expressed as means ± SE (*n *= 3). Ethylene‐treated plants were exposed to an ethylene atmosphere for 24 h, and the bottom three leaves were sampled for AZ tissue (L1, L2, L3; L1 as the oldest). Different letters represent significantly different expression levels (*P *≤ 0.05) between the leaf positions and the sides of the AZ (see Table S1 for details). Asterisk represents significantly different expression levels (*P *≤ 0.05) between the WT and transgenic line. Bottom: Illustration of the location of the leaf petiole AZ on the plant, and sampling sites of the distal and proximal leaf petiole AZ.

Before abscission induction, the expression of *LeACO1* was very low in the leaf petiole AZ samples of both the WT and the transgenic tomato line (Fig. 3). However, *LeACO1* expression increased significantly after ethylene treatment of plants from both lines (Fig. 3, Table S1). After treatment, there were no significant differences in *LeACO1* expression between the two sides of the petiole AZ fracture, and between leaves at different development stages (Fig. 3). However, there was a trend for a gradual increase in *LeACO1* expression in the petiole AZ from the oldest examined (L1) to the youngest (L3) leaf (Fig. 3, Table S1). Additionally, there was a trend to higher *LeACO1* expression on the proximal side of the petiole AZ fracture in the WT plants, which was only seen for the oldest leaf (*i.e*. L1) in the transgenic line (Fig. 3, Table S1).

### Expression of *LeACO4* is relatively high in the tomato leaf petiole abscission zone

**Figure 4:**
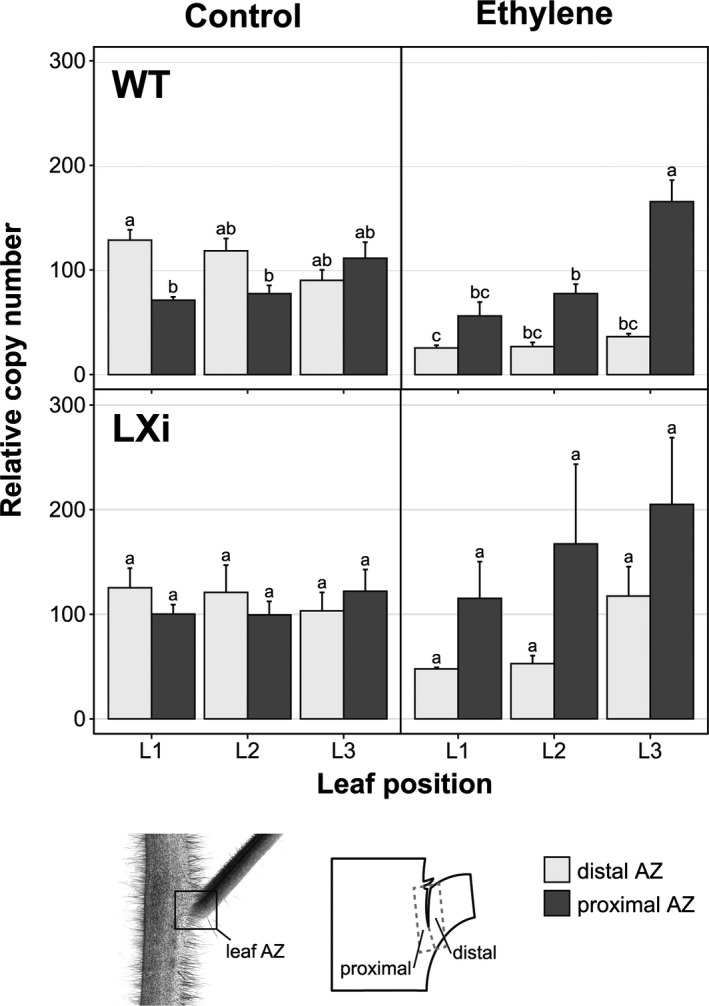
Gene expression profiles of *LeACO4* in the leaf petiole AZ of the ‘VF36’ tomato WT line and transgenic line 1‐7 (LXi) with suppressed *LeLX* expression. Control and ethylene‐treated samples of the distal (white) and proximal (grey) AZ were analysed, with the data expressed as means ± SE (*n *= 3). Ethylene‐treated plants were exposed to an ethylene atmosphere for 24 h, and the bottom three leaves were sampled for AZ tissue (L1, L2, L3; L1 as the oldest). Different letters represent significantly different expression levels (*P *≤ 0.05) between the leaf positions and the sides of the AZ (see Table S1 for details). Bottom: Illustration of the location of the leaf petiole AZ on the plant, and sampling sites of the distal and proximal leaf petiole AZ.

In contrast to *LeACO1*, the initial levels of *LeACO4* expression were high even before abscission induction (Fig. 4). In WT plants there was significantly more *LeACO4* transcript on the distal side of the petiole AZ fracture in the older L1 and L2 leaves, in comparison with the youngest L3 leaf (Fig. 4). A similar trend was observed in the transgenic line (Fig. 4, Table S1). In all of the WT leaves examined, *LeACO4* expression dropped significantly 24 h after abscission induction with the ethylene treatment on the distal side of the petiole AZ fracture (Fig. 4, Table S1), with a similar trend also seen in the transgenic plants (Fig. 4, Table S1). In both the WT and transgenic plants, *LeACO4* expression was higher on the proximal side of the petiole AZ fracture in the L3 leaves (Fig. 4).

### Localisation of ACO1 to the vascular tissues

Although the antibodies used in this study were raised against the ACO protein sequence from *Arabidopsis* that shares 82% amino acid identity with ACO1 from tomato, some overlapping with tomato ACO4 (66% amino acid identity) could not be totally excluded. However, the Western blot revealed very high specificity of these antibodies to ACO1 (Figure S2).

**Figure 5:**
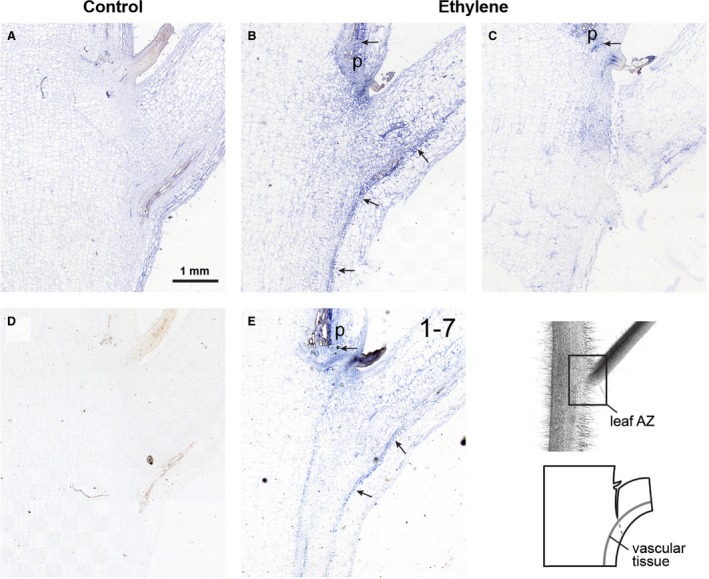
Immunolocalisation of the ACO1 protein in the leaf petiole AZ. (A) Control AZ before abscission induction. (B), (C) AZ of the WT plants 24 h after abscission induction by ethylene treatment in a young leaf (L3) in which the fracture has not yet formed (B), and in an old leaf (L1) in which the fracture is complete (C). (D) Immunolocalisation control using non‐immune serum. (E) AZ of the transgenic line 1‐7 24 h after abscission induction by ethylene treatment and before fracture formation. Bottom, right: Illustration of the leaf petiole AZ and location of the vascular tissue on the plant. Arrows indicate the presence of the immunolocalisation signal; p, leaf primordium.

In agreement with the increased amount of *LeACO1* transcript in the AZ after abscission induction, in the same time period the ACO1 protein was detected. In the AZ of the WT petiole of young leaves in which the separation fracture had not started to form (Fig. 5B), ACO1 protein was immunolocalised mainly to the vascular tissues that traversed the leaf petiole AZ (Fig. 5B). In addition, there was also some visible signal dispersed throughout the whole AZ (Fig. 5B). Of note, the immunolocalisation signal became fainter from young leaves to the oldest leaf (L1), with a fully expanded fracture inside the AZ (Fig. 5C). In the L1 leaves there was almost no ACO1 signal in the vascular tissues, but some residual signal was still detected in the proximal side of the AZ (Fig. 5C). In addition, the immunolocalisation signal was also detected in leaf primordia (Fig. 5B, C), where the expression of *LeACO* genes has also been reported (http://gbf.toulouse.inra.fr/tomexpress/www/welcomeTomExpress.php).

A similar pattern of expression was also detected in the AZ of petioles in the transgenic line (Fig. 5E). However, besides the vascular localisation, an additional immunosignal of ACO protein was dispersed in those plants, but was more concentrated along the line of future fracture formation (Fig. 5E).

## Discussion

We have previously shown the spatial distribution of cell separation and PCD, as two developmental processes that take place in the petiole AZ. While in the AZ of the leaf petioles of the WT plants 24 h after abscission induction, expression of polygalacturonase genes associated with cell separation, *LeTAPG1* and *LeTAPG4*, and induction of TAPG4 protein are limited to the proximal side of the AZ (Fig. 1; Lumpert 2010; Bar‐Dror *et al*. 2011), the *LeLX* expression associated with PCD and the ultrastructural features of PCD are located primarily on its distal side (Figs 2, S1; Bar‐Dror *et al*. 2011). After abscission induction in plants of the 1‐7 transgenic line with inhibited *LeLX* expression and reduced LX protein level in which both leaf petiole and flower pedicel abscission are significantly inhibited (Lers *et al*. 2006; Bar‐Dror *et al*. 2011), at least some TAPG4 was present in the AZ (Fig. 1). However, in these plants the fracture formation with cell separation finally occurred, although with a delay compared to that of the WT plants. Of note, the cells from both sides of the AZ in transgenic plants did not differ significantly from those before the abscission induction (Fig. 2). Specifically, we were not able to detect either features of intensive membrane trafficking or PCD‐related features that are hallmarks of the WT AZ. Since tomato plants with reduced LX protein levels develop without major phenotypic alteration under optimal growth conditions (Lers *et al*. 2006), it is possible that LX is involved through different PCD processes or in a cellular recycling pathway that functions in response to environmental stress conditions (Zhou *et al*. 2013).

Our data demonstrate the alternating expression of *LeACO1* and *LeACO4* during leaf petiole abscission (Figs 3, 4). The expression of these *LeACO* genes also exhibited some spatial asymmetry, which has previously been shown for cell separation and PCD (Bar‐Dror *et al*. 2011). Induction of *LeACO* genes showed dependence on development stage of the leaf: the younger the leaf, the more time required for induction by ethylene. It has been shown that older leaves at the base of the tomato plant are more prone to abscission (Burg 1968). Additionally, it was suggested that an important step in the abscission process is the gain of ethylene sensitivity in the AZ (Meir *et al*. 2010). After 24 h of ethylene treatment, the fracture in the AZ had already formed in the oldest leaves, and the process gradually spread to the younger leaves (Fig. 5B, C). Similar leaf‐age dependence has also been shown for induction of LX protein with external ethylene (Lers *et al*. 2006) and it was suggested that the phenomenon might be explained by differences in ethylene sensitivity of the AZ in different aged leaves.

It is worth noting that the ACO1 protein was localised to the vascular tissues traversing the leaf petiole AZ (Fig. 5). In comparison, the previous laser microdissection analysis of the AZ of the tomato flower pedicel revealed high *LeACO1* expression in the vascular tissues spread throughout both sides of the AZ, and low expression in cell rows in which the fracture actually formed. The analysis also revealed that the ACO1 protein is localised to the same area (Chersicola *et al*. 2017). In that study, ACO1 was found mainly in the phloem companion cells, which was thus suggested as the side for synthesis of ethylene. An additional striking outcome of that study was that cell separation and PCD also share a common origin in the vascular tissues inside the flower pedicel AZ (Chersicola *et al*. 2017). On the other hand, in the tomato petiole AZ the spatial relationships between ACO1, cell separation and PCD are less clear, as the formation of the abscission fracture, and thus cell separation, start in the part of the AZ just opposite the vascular tissues that traverse the petiole (this study; Bar‐Dror *et al*. 2011; Dermastia *et al*. 2012). However, in the petiole AZ of the transgenic line, the signal of ACO1 was also clearly localised to the side of the future fracture, but in the WT it was detected there only when the fracture had already formed (Fig. 5). At the moment, it is not known whether these processes are associated with several diffusible signals derived from vascular tissue that are required for abscission, as previously suggested (Thompson & Osborne 1994; McManus 2008; Tucker & Yang 2012).

Additionally, we examined whether the transcript abundances of *LeACO1* and *LeACO4* vary during leaf abscission in the 1‐7 transgenic line that shows inhibited *LeLX* expression (Figs 3, 4). The results of this study showing similar expressions of *LeACO1* and *LeACO4* in WT and transgenic plants suggest that the expression of these *LeACO* genes is not affected by suppression of *LeLX* expression that is involved in PCD in the leaf petiole AZ, although both genes are induced by ethylene.

## Supporting information


**Table S1.** Statistical analyses of differences in expression of the *LeACO1* and *LeACO4* genes.

**Fig. S1.** Gene expression profiles of *LeLX* in the leaf petiole abscission zone (AZ) of WT tomato line VF36 and tomato lines with changed expression of *LeLX* (LXi).

**Fig. S2.** Western blot of the proteins extracted from the flower pedicels before and after abscission induction, using ACC oxidase antibodies.
