# Supplementary material for: The pattern of 1‐aminocyclopropane‐1‐carboxylate oxidase induction in the tomato leaf petiole abscission zone is independent of expression of the ribonuclease‐LX‐encoding LeLX gene
Source: Plant Biol (Stuttg). 2018 Apr 26;20(4):722–8. doi: 10.1111/plb.12730 (PMC6032998; doi:10.1111/plb.12730)
Supplement: Supplementary file 1 — Table S1. Statistical analyses of differences in expression of the LeACO1 and LeACO4 genes. [file PLB-20-722-s001.pdf]

The pattern of 1-aminocyclopropane-1-carboxylate oxidase induction in the tomato leaf petiole abscission zone is independent of expression of the ribonuclease-LX-encoding *LeLX* gene

Marko Chersicola, Aleš Kladnik, Magda Tušek Žnidarič, Amnon Lers and Marina Dermastia

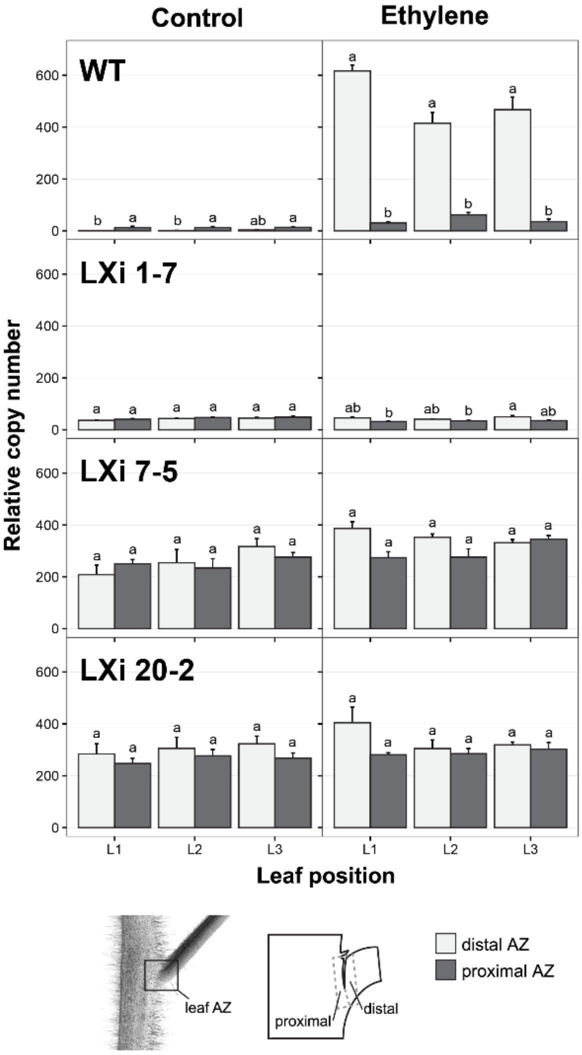

Figure S1. Gene expression profiles of *LeLX* in the leaf petiole abscission zone (AZ) of WT tomato line VF36 and tomato lines with changed expression of *LeLX* (LXi) (Lers *et al.* 2006). Control and ethylene-treated samples of the distal (white) and proximal (gray) AZ were analyzed, with the data expressed as means ± SE (n=3). Ethylene treated plants were exposed to ethylene atmosphere for 24 h and the bottom three leaves were sampled (L1, L2, L3, where L1 is the oldest leaf). Different letters represent significantly different expression levels ($p \leq 0.05$) between the leaf positions and the sides of the AZ. Below: The location of the leaf AZ on the plant and identification of sampling sites of the distal and proximal leaf AZ.
