# Supplementary material for: The pattern of 1‐aminocyclopropane‐1‐carboxylate oxidase induction in the tomato leaf petiole abscission zone is independent of expression of the ribonuclease‐LX‐encoding LeLX gene
Source: Plant Biol (Stuttg). 2018 Apr 26;20(4):722–8. doi: 10.1111/plb.12730 (PMC6032998; doi:10.1111/plb.12730)
Supplement: Supplementary file 2 — Fig. S1. Gene expression profiles of LeLX in the leaf petiole abscission zone (AZ) of WT tomato line VF36 and tomato lines with changed expression of LeLX (LXi). [file PLB-20-722-s002.pdf]

The pattern of 1-aminocyclopropane-1-carboxylate oxidase induction in the tomato leaf petiole abscission zone is independent of expression of the ribonuclease-LX-encoding *Le*LX gene

Marko Chersicola, Aleš Kladnik, Magda Tušek Žnidarič, Amnon Lers and Marina Dermastia

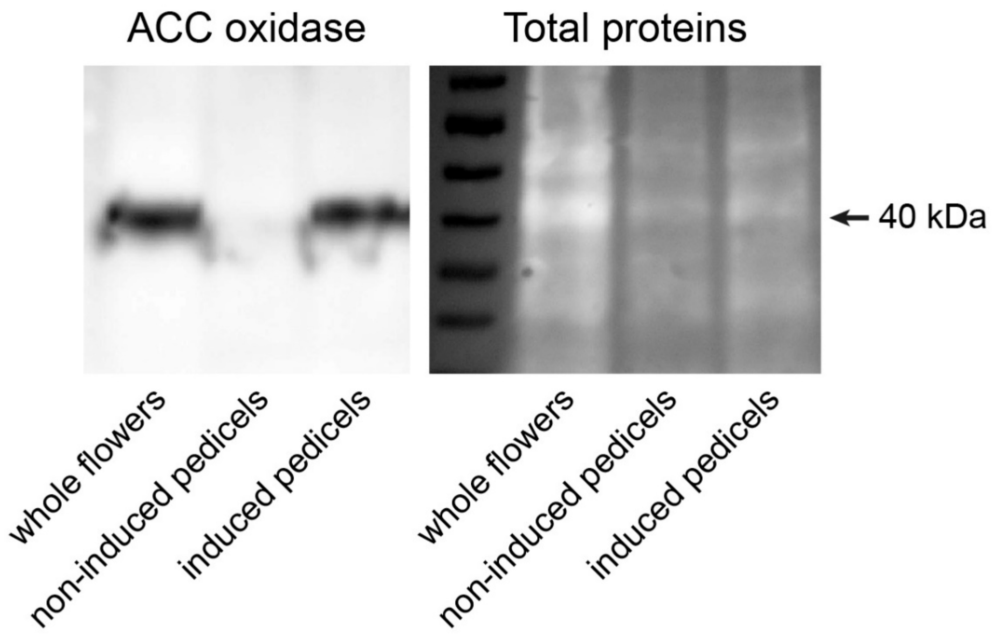

Figure S2. Western blot of the proteins extracted from the flower pedicels before and after abscission induction, using ACC oxidase antibodies (Santa Cruz Biotechnology aN-19) shows no signal in pedicels before abscission (non-induced pedicels), but a strong signal in pedicels 12 h after abscission induction (induced pedicels). The signal corresponds to the visible band at 40 kDa in the total proteins image. In relation to the *LeACO* expression data (Chersicola et al. 2017) this indicates that the ACCO oxidase antibodies are specific for ACO1, but not for ACO4. *LeACO1* is induced in tomato flower pedicel only after abscission induction, while the *LeACO4* expression level is high in flower pedicels before abscission induction and then rapidly declines after induction. *LeACO1* is not expressed in flower pedicels before the induction (Chersicola et al., 2017).

Materials and methods

Wildtype tomato VF-36 plants were used to evaluate ACO antibody specificity. Fresh open flowers at anthesis were cut from the plant as inflorescences on stems and kept in water until sampling. Samples before abscission induction were separated into flowers and pedicels and immediately frozen in liquid nitrogen. Abscission in pedicel was induced by removing the flowers at their base. Induced pedicels were sampled 12 h after induction. All samples were frozen in liquid nitrogen and stored at -80 °C until protein extraction. Approximately 100 mg of tissue was homogenized in 100 µl of extraction buffer (25 mM Tris-HCl pH 6.8, 150 mM NaCl, 10 mM EDTA, 10% glycerol, 1% Triton X-100, 2 mM DTT and 1% protease inhibitor) using Fastprep-24 Sample Preparation Instrument (MP Biomedicals). 2x sample buffer (125 mM Tris-HCl pH 6.8, 4% SDS, 20% glycerol, 0.02% bromophenol blue and 200 mM DTT) was added and samples were boiled for 5 minutes. SDS-PAGE was performed with Bio-Rad Mini-PROTEAN TGX Stain-Free Precast 4-15% gel with PageRuler Prestained Protein Ladder, 10 to 180 kDa (ThermoFisher). Proteins were transferred onto PVDF membrane and the fluorescence of total proteins on the membrane was detected using UVP ChemStudio PLUS Imaging System (Analytik Jena). Membrane was blocked with 5% non-fat dry milk (Biorad) in TBST (0.1% Tween-20 in TBS) for 2 h and incubated with 1:1000 ACC oxidase antibodies (Santa Cruz Biotechnology aN-19) overnight at room temperature with shaking. After incubation, membrane was washed four times in TBST, incubated with 1:5000 anti-goat IgG-HRP (Abcam) for 1 h and washed six times in TBST. Chemiluminescent signals were detected using Amersham ELC Prime Western blotting detection reagent and visualized with UVP ChemStudio PLUS Imaging System (Analytik Jena).
